# Noninvasive Prenatal Paternity Testing: A Review on Genetic Markers

**DOI:** 10.3390/ijms26104518

**Published:** 2025-05-09

**Authors:** Laura Carrara, Diana Hall

**Affiliations:** 1 School of Criminal Justice, Faculty of Law, Criminal Justice and Public Administration, University of Lausanne, Batochime, 1015 Lausanne, Switzerland; laura.carrara@unil.ch; 2 Forensic Genetics Unit, University Center of Legal Medicine, Lausanne-Geneva, Lausanne University Hospital and University of Lausanne, 1000 Lausanne, Switzerland

**Keywords:** Noninvasive prenatal paternity testing (NIPPT), cell free fetal DNA (cffDNA), forensic genetics, DNA mixture, pregnancy

## Abstract

Noninvasive prenatal paternity testing (NIPPT) is a crucial tool in forensic contexts, particularly in cases involving post-rape pregnancies. It enables judicial authorities and victims to promptly address these situations by determining the paternity of the fetus within a few weeks of pregnancy. NIPPT relies on the analysis of cell-free fetal DNA (cffDNA) found in the maternal bloodstream. However, the abundance of maternal DNA presents a significant challenge in detecting fetal DNA. As a result, research has focused on improving methods for isolating or enriching fetal DNA and, specifically in the context of forensic genetics, on the development of suitable genetic markers. The use of Single Nucleotide Polymorphisms (SNPs) along with novel compound markers or composite multiplexes, has shown promising results. Despite significant advances, partly driven by the increased use of Massive Parallel Sequencing (MPS), challenges remain in validating markers-based NIPPT assays for forensic casework. Further studies are required to enhance the sensitivity of these tests, particularly during the early stages of pregnancy, such as the first trimester. Additionally, improving and standardizing statistical frameworks for result evaluation and interpretation is essential to ensure compatibility with forensic standards.

## 1. Introduction

In the late 1990s, Lo et al. discovered the presence of cell-free fetal DNA (cffDNA) circulating in the blood of pregnant women [1]. This DNA can be detected as early as the sixth week of gestation and results from trophoblast apoptosis during placental development [2,3]. The process continues throughout pregnancy, leading to a progressive increase in cffDNA levels [4]. Depending on the gestational week, the proportion of cffDNA in the maternal plasma ranges from 2 to 20%, with the remaining DNA originating from the mother [4,5,6]. Within a few hours after delivery, cffDNA is cleared from maternal blood, and therefore does not interfere with cffDNA detection in subsequent pregnancies [7].

The discovery of cffDNA initially revolutionized the field of fetal medicine by providing the foundation for noninvasive fetal DNA analysis. New methods, based on venous blood sampling from the mother, eliminated the risks of miscarriage associated with other more invasive procedures such as amniocentesis [8,9] and chorionic villus sampling [10,11], while also allowing cffDNA analysis at an earlier stage of pregnancy. Progress in cffDNA collection procedures has encouraged the development of new techniques for prenatal diagnostics and the advent of noninvasive prenatal paternity tests (NIPPT).

In forensic genetics, NIPPT is essential in various contexts, including unintended pregnancies resulting from rape. The analysis of cffDNA can play a crucial role in helping judiciary authorities establish a link between the victim and the potential aggressor by determining the paternity of the fetus even before birth. Although post-rape pregnancies have been consistently reported [12,13,14,15,16], accurately estimating the number of such cases is challenging due to underreporting and limited epidemiological data. Studies conducted in the US suggest that 4–10% of rapes may lead to an unintended pregnancy [14,15,16,17,18]. However, this rate is likely an underestimate [13] and may be much higher in armed conflict zones [19]. This situation can have profound medical and psychological implications for the victims [12,15,19] and is therefore important that medico-legal professionals have access to the appropriate resources to assist them.

While many private diagnostic centers and laboratories already offer NIPPT as a service, several issues must be addressed before these methods can be reliably implemented in forensic laboratories. One of the primary difficulties stems from the mixed nature of the plasma sample, where the analytical challenge lies in accurately targeting fetal DNA while minimizing the interference of maternal DNA. This often results in methods lacking the sensitivity required for accurate detection, particularly in the early stages of pregnancy.

**Figure 1:**
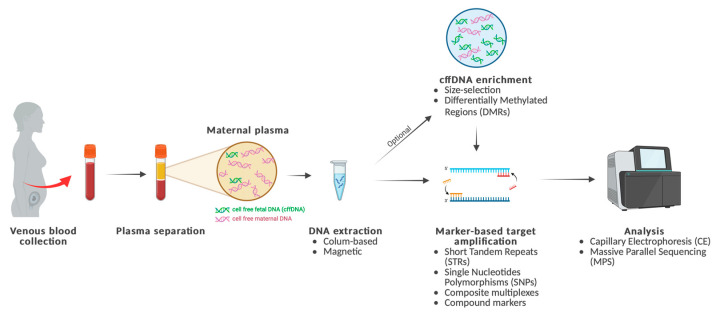
Main steps in cell free fetal DNA (cffDNA) analysis for Noninvasive prenatal paternity testing (NIPPT), detailing the key method developed for DNA extraction, cffDNA enrichment, markers-based target amplification and analysis. (Created in BioRender. Carrara, L. (2025) https://BioRender.com/hp8f10f, accessed on 8 May 2025).

Despite increasing research in cffDNA analysis, no protocol has been fully standardized. Several approaches have been explored, which can be categorized into three main areas: extraction methods, cffDNA enrichment techniques, both of which benefit prenatal diagnostics and paternity testing, and genetic markers studies specific to NIPPT. Various methods have been implemented within each category (Figure 1), most of them have been comprehensively detailed in Zhang et al. [20].

In summary, extraction methods are predominantly column-based [21] or magnetic [22,23]. Enrichment strategies exploit the differences between cffDNA and maternal DNA fragments. They can be applied to selectively increase cffDNA concentrations in plasma [24,25]. The most common are selection of DNA fragments based on their size [26], which is typically shorter for cffDNA (<300 bp) [27], or selection based on Differentially Methylated Regions (DMRs) [28]. The analysis of cffDNA for NIPPT relies on target amplification, which can be performed using different genetic markers such as Short Tandem Repeats (STRs), Single Nucleotide Polymorphisms (SNPs), compound markers, and composite multiplexes. The analysis is generally performed using Capillary Electrophoresis (CE) or Massive Parallel Sequencing (MPS).

This review aims to provide an updated overview of the current NIPPT protocols based on genetic markers, offering a comprehensive framework for evaluating their performance and limitations. We discuss the challenges in cffDNA analysis and results interpretation, additionally we propose potential research directions for the development of NIPPT methods that can be validated for forensic applications.

## 2. Methodology

This literature review included articles reported in a previous review [20] as well as all additional peer-reviewed, English-language studies focusing on the development and validation of genetic markers for NIPPT since the discovery of cffDNA in 1997. Studies addressing data analysis and result interpretation related to NIPPT applications were also retained. However, articles on prenatal diagnostic methods, nonspecific to NIPPT, were excluded, as were studies focusing on the development and validation of new markers sets potentially applicable to NIPPT but not empirically tested on maternal plasma, and studies on postnatal paternity testing. Literature searches were conducted using PubMed with the keywords “NIPPT”, “NIPAT”, “Noninvasive/Non-invasive prenatal paternity test”, “Paternally inherited alleles” along with “Forensic”. Based on the criteria described, a comprehensive list of papers was identified and discussed (*n* = 33).

## 3. Application of Genetic Markers in NIPPT

### 3.1. Short Tandem Repeats (STRs)

STRs are the genetic markers of choice for forensic identification. These polymorphisms are genomic regions characterized by varying numbers of two- to five-nucleotide repeat sequences and can be found on both autosomal and sex chromosomes. Due to their high degree of polymorphism and extensive validation in forensic genetics, STRs were among the first markers considered for cffDNA analysis (Table 1).

One of the earliest studies evaluating the potential of STR markers for cffDNA detection was conducted by Birch et al. [29] in 2005. The research group utilized the ampFLSTR^TM^ SGM Plus^TM^ Kit (Thermo Fischer Scientific, Waltham, MA, USA) for capillary electrophoresis (CE) analysis on two types of samples: maternal plasma from five confirmed male pregnancies and five confirmed female pregnancies (gestational ages: 29–41 weeks), and in vitro simulated DNA mixtures of male and female DNA at varying ratios (1:2, 1:660). The results, limited to the sex determination marker amelogenin, revealed that fetal DNA amplification occurred solely in 1:2 DNA mixtures while in maternal plasma samples, amelogenin was detectable only starting from the 38th week of pregnancy. In 2009, Wagner et al. [30] published a study on the application of a commercial STRs panel for the analysis of cffDNA. The ampFLSTR^TM^ Identifiler^TM^ Kit (Thermo Fischer Scientific) was used for CE analysis of 20 plasma samples from pregnant women at gestational ages between 9 and 29 weeks. The study reported inconsistent amplification of autosomal loci from fetal DNA, likely due to inhibited primer binding caused by excess maternal DNA. The authors concluded that the amount of cffDNA present in maternal plasma was insufficient for the reliable identification of autosomal fetal alleles. In the same year, Tang et al. [31] tested 16 STR markers using the AmpFlSTR^TM^ Profiler Plus PCR Amplification Kit (Thermo Fischer Scientific) for CE analysis of 47 plasma samples from women at gestational ages ranging from the 8th to the 37th week of pregnancy. The study reported detection rates for paternally inherited fetal alleles of 66.67%, 85.71%, and 94.12% in the first, second, and third trimesters, respectively. However, caution should be exercised when interpreting these results due to the very small sample size in the first trimester (*n* = 3) and to the high number of PCR cycles used (i.e., 40).

While STR markers are a cornerstone in forensic genetics, these studies highlighted their limited performance in cffDNA analysis, especially in the first trimester of pregnancy, where detection rates were often insufficient for paternity determination.

To address these challenges, some researchers have explored a particular type of STR markers, mini-STRs, that are specifically designed for the analysis of short DNA fragments. These markers, combined with Massive Parallel Sequencing (MPS), are considered more suitable for short cffDNA fragments, because primers are designed to reduce the size of the flanking region around the STR [32]. In 2015, Gysi et al. [33] published a preliminary study comparing the performance of a conventional STR kit (GlobalFiler^TM^ PCR Amplification Kit, Thermo Fischer Scientific) analyzed by CE and an MPS panel targeting mini-STRs (Ion AmpliSeq HID STR 25-plex, Thermo Fischer Scientific). In this pilot study, they analyzed plasma samples from two pregnant women at 10 and 16 weeks of pregnancy. Although the GlobalFiler^TM^ kit detected 4 paternal alleles out of 21 in both pregnancies, the remaining alleles were either masked by maternal DNA or dropped out. When analyzed with the Ion AmpliSeq HID STR 25-plex kit, 9 and 12 paternal alleles were detected at 16 and 10 weeks of pregnancy, respectively, and the rest were likely masked by maternal alleles. No drop-out was confirmed, and paternity was successfully established in both cases without any observed drop-ins. This study suggested that mini-STRs analyzed via MPS outperform conventional STRs analyzed by CE. However, a small sample size prevented generalization of the conclusions. In 2022, Song et al. [34] applied a panel of 23 mini-STRs to MPS analysis of 28 plasma samples collected from pregnant women between the 12th and 38th weeks of gestation (18 samples were from the first trimester). The study reported a matching probability of 75–100% when comparing fetal profiles to paternal reference profiles, and no correlation with pregnancy stage was observed. This pilot study further demonstrated that mini-STRs analyzed via MPS performed better than conventional STRs in NIPPT cases.

Despite the recent improvements, autosomal STRs have never been the preferred method for cffDNA analysis, with only a limited number of studies reporting their routine implementation and application in NIPPT caseworks [35,36]. To overcome their limitations, some researchers have explored the use of Y chromosome-specific STR markers (Table 1). Although these markers are not applicable in cases involving female fetuses, and are limited to determining the paternal lineage, they have demonstrated an increased performance compared to autosomal STRs.

In 2006, Deng et al. [37] published a study applying a Y-STRs multiplex panel, comprising nine markers, to the CE analysis of 64 plasma samples from pregnant women, including 6 samples from the first trimester. Among the participants, 30 women were carrying a male fetus. The analysis yielded an average of 7.3 paternal alleles per sample, with the probability of detecting Y-STRs alleles ranging from approximately 67 to 93%, depending on the loci examined. No false positives were observed in plasma samples from mothers carrying a female fetus. In the previously mentioned study by Wagner et al. (2009) [30] on autosomal STRs, the authors also tested the commercial ampFLSTR^TM^ Yfiler^TM^ Kit (Thermo Fischer Scientific) using CE. The results showed that 6–16 (complete profile) fetal alleles matching the paternal DNA profile could be successfully amplified. However, false positive rates were not assessed. Barra et al. (2015) [38] conducted a study on 20 plasma samples from pregnant women (between 12 and 36 weeks of pregnancy, including 13 from the first trimester) using the PowerPlex^®^ Y23 Kit (Promega Corporation), the ampFLSTR^TM^ Yfiler^TM^ Kit (Thermo Fischer Scientific), and two in-house developed multiplexes targeting 27 Y markers, all analyzed by CE. Using 50 PCR cycles, they successfully amplified 22–27 markers per case. The resulting profiles achieved a complete match with the paternal profile in 16 out of the 20 cases.

Similar to autosomal STRs, a shortened version of Y-STRs, known as Y-chromosome mini-STRs, was developed to enhance the amplification of cffDNA. In 2022, Song et al. [39] published a study on the application of a 12 Y-chromosome mini-STRs panel to the MPS analysis of 24 plasma samples from pregnant women between the 21st and the 37th weeks of pregnancy. Of these, 14 were male and 10 were female. Using this method, paternity could be correctly attributed for 13 male fetuses. The probability of paternity ranged from 98.3 to 99.9%. For the remaining cases, a probability of 14.9% was obtained due to a mutation at one locus.

**Table 1:** Summary of studies on genetic markers contributing to the development of Noninvasive prenatal paternity testing (NIPPT).

Type of Marker	Panel	Number of Markers Analyzed	Technology	Gestational Age (Weeks)	Total Number of Samples	Results Evaluation	Reference
Autosomal Short Tandem Repeats (a-STRs)	ampFLSTR^TM^ SGM Plus^TM^ Kit *	11	PCR-based method	29–41	10	N/A	Birch et al. [29]
	AmpFlSTR ^TM^ Profiler Plus PCR Amplification Kit *	16	CE (310 Genetic Analyzer *)	8–37	47 (1st Tri: 6)	N/A	Tang et al. [31]
	GlobalFiler™ PCR Amplification Kit */early access version of Ion AmpliSeq HID STR 25-plex *	21/24	CE (310 Genetic Analyzer *) and MPS (Ion Torrent PGM™ *)	10; 16	2 (1st Tri: 1)	Posterior probability	Gysi et al. [33]
	Custom panel	23	MPS (NextSeq 500 system **)	12–38	28 (1st Tri: 18)	Matching probability	Song et al. [34]
Y-chromosome Short Tandem Repeats (Y-STRs)	Custom assay	9	CE (ABI Prism™ 377 DNA Sequencer) *	ND	64 (1st Tri: 6)	N/A	Deng et al. [37]
	ampFLSTR Yfiler™ */PowerPlex^®^ Y23 *** and two custom panels	67 (17/23/27)	CE (3500 Genetic Analyzer *)	12–36	30 (1st Tri: 22)	CPI and posterior probability	Barra et al. [38]
	Custom panel	12	MPS (NextSeq 500 system **)	21–37	24 (1st Tri: 0)	CPI and posterior probability	Song et al. [39]
a-STRs and Y-STRs	ampFLSTR^TM^ Identifiler^TM^ */ampFLSTR Yfiler™ *	31 (15 STRs + 16 Y-STRs)	CE (310 Genetic Analyzer *)	9–29	20	ND	Wagner et al. [30]
	Three custom panels	34 (12/10/12)	MPS (Ion Torrent PGM *)	≥9	>180	CPI	Whittle et al. [36]
Single Nucleotide Polymorphisms (SNPs)	Custom panel	92	PCR-based method	ND	154	N/A	Tynan et al. [40]
	Custom panel	384	PCR-based method	8–14	30	Probability of exclusion	Guo et al. [41]
	Microarray (HumanCytoSNP-12 array **)	300,000	Illumina Infinium technology **	6–21	21 (1st Tri: 11)	Accuracy (Diagnostic potency)	Ryan et al. [42]
	Three custom panels	5000 to 8000	MPS (Hiseq™ 2000 **)	13–30	17 (1st Tri: 0)	CPI	Jiang et al. [43]
	Custom panel	1479	MPS (Hiseq™ 4000 **)	11–18	7 (1st Tri: 3)	ND	Yang et al. [44]
	Custom panel	720	MPS (Ion Torrent PGM *)	9–21	20 (1st Tri: 11)	ND	Yang et al. [45]
	Custom panel	1795	MPS (Hiseq™ 2000 **)	9–21	34 (1st Tri: 11)	CPI	Qu et al. [46]
	Precision ID Identity Panel *	124	MPS (Ion S5™ System *)	4–20	15 (1st Tri: 15)	Paternity Index	Christiansen et al. [47]
	Custom panel	4151	MPS (BGISEQ-500, MGI)	6–35	358 (1st Tri: 155)	CPI	Chang et al. [48]
	Custom panel	356	MPS (MiniSeq™ **)	7–20	15 (1st Tri: 10)	CPI and posterior probability	Tam et al. [49]
	Custom panel	5226	MPS (Hiseq™ 2000 **)	9–18	15 (1st Tri: 11)	CPI	Xie et al. [50]
	Custom panel	861	MPS (Ion S5™ System *)	ND (1st Tri)	9 (1st Tri: 9)	CPI	Giannico et al. [51]
Short Tandem Repeats (STRs) and SNPs	ForenSeq^TM^ DNA Signature Prep ****	152 (94 SNPs + 58 STRs)	MPS (MiSeq FGx ****)	7–24	17 (1st Tri: 8)	CPI	Shen et al. [52]
Microhaplotypes (MHs)	Custom panel	60	MPS (Hiseq™ 2000 **)	6–13	15 (1st Tri: 15)	CPI	Ou and Qu [53]
	Custom panel	60	MPS (Hiseq™ 2000 **)	≥10	19	CPI	Bai et al. [54]
	Custom panel	15	CE (3130 Genetic Analyzer *)	>18	26 (1st Tri: 0)	ND	Zhang et al. [55]
Deletion/Insertion Polymorphisms with STRs (DIP-STRs)	Custom panel	28	CE (3500xl Genetic Analyzer *)	10–39	48 (1st Tri: 27)	ND	Moriot et al. [56]
	Custom panel	47	CE (3500xl Genetic Analyzer *)	7–13	87 (1st Tri: 87)	CPI and posterior probability	Damour et al. [57]
DIP-STRs and SNP-STRs	Custom panel	17 (6 DIP-STRs + 11 SNP-STRs)	CE (3130 and 3500 Genetic Analyzers *)	>18	21 (1st Tri: 0)	ND	Tan et al. [58]

* Thermo Fischer Scientific^®^ (Waltham, MA, USA)/** Illumina^®^ Inc. (San Diego, CA, USA)/*** Promega^®^ (Madison, WI, USA)/**** Verogen^®^ (San Diego, CA, USA). CE = Capillary Electrophoresis/MPS = Massive Parallel Sequencing/N/A = not applicable/ND = not determined/1st Tri = first trimester/CPI = combined paternity index.

### 3.2. Single Nucleotide Polymorphism (SNPs)

Among the various markers used in the development of NIPPT, SNPs (Single Nucleotide Polymorphisms) markers, have been widely reported [40,41,42,43,44,45,46,47,48,49,50,51,59] (Table 1). SNPs markers offer several advantages over STRs. For instance, their mutation rate is lower [60], reducing the risk of mutation between father and child, making them particularly suitable for paternity testing [61]. Furthermore, SNPs analysis does not generate amplification artifacts, such as stutters, which simplifies the result interpretation [62]. Most importantly, the small size of SNPs, makes them well suited for the analysis of cffDNA.

Tynan et al. (2011) [40] were among the first to report the potential application of SNP markers for detecting paternally inherited alleles in maternal plasma. However, their study primarily focused on prenatal diagnostics rather than NIPPT. Their approach involved the detection of cffDNA using restriction enzymes. By applying a panel of 92 SNPs, they detected 5–21 informative SNPs across 103 plasma samples. Guo et al. (2012) [41] published a study on the application of a 384 SNPs panel to analyze 30 plasma samples collected from pregnant women between 8–14 weeks of gestation. The authors previously selected case-specific informative markers (17 on average) and used a PCR-CE-based analytical method. The results showed that paternity could be correctly determined for all 30 cases analyzed. However, they reported a false positive rate of 13.3% for fetal DNA amplification and a drop-out rate of 2.7%. In 2013, Ryan et al. [42] proposed a novel method based on an SNP array (HumanCytoSNP-12 array, Illumina) that allowed for the simultaneous analysis of 300,000 SNPs. They tested 21 samples from pregnant women between the 6th and the 21st week of pregnancy (11 from the first trimester). The authors used an informatics method called the Parental Support algorithm to compare the results obtained on plasma samples, with the genotypes of the confirmed father as well as 1820 unrelated individuals. This method calculates a test statistic for each mother and alleged father to evaluate the compatibility between the tested father’s genotype and the fetal component of the genotype observed in the maternal plasma. The distribution of test statistics was first observed using data from unrelated men. The results for alleged fathers were then tested against this distribution, if a result fell within it, the alleged father was excluded, if it fell outside, he was considered the biological father. Using this method, the paternity was successfully determined for 20 out of 21 cases, with 1 sample excluded due to insufficient cffDNA quantity. The *p* values for the comparison with real fathers were <10^−4^ for all tested samples (100%). Although the test seems promising, it raises some concerns regarding its forensic application. First, some of the SNPs included in the kit are related to human diseases. Second, the paternity assignment method used in this study is not compatible with the International Society of Forensic Genetics (ISFG) and Paternity Testing Commission (PTC) recommendations, which led to criticism from Drabek et al. (2014) [63]. Ryan et al. (2014) [64] defended their approach, stating that the interpretation approach proposed by Drabeck et al. [63] did not consider the specificity of NIPPT, which involves the analysis of cffDNA mixed with high quantities of maternal DNA.

In 2016, Jiang et al. [43] published a pilot study on the development of NIPPT using SNPs markers analyzed using MPS. To the best of our knowledge, this is the first study of its kind to use this technology. The authors analyzed two types of samples: plasma samples from pregnant women in the second and third trimesters (*n* = 17), which were analyzed by MPS using an SNPs panel containing 5000 to 8000 SNPs, and amniocentesis samples, which were analyzed with an STRs panel by CE. They developed a new algorithm for PI calculations, based on a Bayesian model that integrates the probability of each possible fetal genotype and considers the probability of sequencing depth distribution in maternal plasma. Only SNPs homozygous in the mother samples were included in the CPI calculations. By comparing the CPI values obtained from both sample types, the authors concluded that the MPS method was as accurate as the invasive method and successfully determined paternity in all cases. However, they noted that higher sequencing depth (up to 200×) would be required for cases where the fetal fraction is below 3.5%. Following this pilot study, several other studies were published on the development of custom SNPs assays for NIPPT, using various MPS platforms [44,45,46,48,50,51]. These studies typically analyzed a large number of SNPs, ranging from hundreds [45,51] to thousands [44,46,48,50], sometimes achieving exceptionally high CPI values [46,48,51]. Most of the studies reported that among the SNPs loci tested, only a proportion (typically those homozygous in the maternal genotype and being over a predefined coverage threshold) were considered informative. Additionally, despite the high-throughput sequencing, which allows for high sensitivity, many paternal alleles within the informative markers were filtered out because they were below the allelic detection threshold, thus reducing the number of effective loci available for paternity determination. Among these studies, it is worth mentioning the study of Giannico et al. [51], testing a panel of 861 SNPs. This study was characterized by the application of the NIPAT-flow algorithm for data analysis. The algorithm infers the fetal genotype based on the genotype determined using maternal plasma samples. It compares these genotypes to different alleged fathers and produces a final report including the paternity probability for each comparison. PI are then calculated using a likelihood ratio formula adapted to NIPPT, which accounts for parameters such as sequencing errors and de novo mutations, and is optimized for samples containing a low fetal fraction. The algorithm demonstrated high performance, even when comparing the genotypes of simulated full brothers, achieving a false positive rate close to zero.

In 2019, Christiansen et al. [47] tested a reduced SNPs panel developed explicitly for forensic human identification, the Precision ID Identity Panel (Thermo Fisher Scientific). The assay targeted 124 SNPs and was analyzed using MPS on 15 plasma samples collected from pregnant women at various time points between the 4th and 20th weeks of pregnancy (4, 7, 12, 16, and 20 weeks). The results indicated that paternally inherited alleles were detectable in a limited number of cases starting from the seventh week of pregnancy, with all cases showing detectable alleles by the 12th week. The number of detected alleles increased progressively through the pregnancy, with a median range of 0 to 12 across the five time-points tested. The mean PI ranged from 24 at week 12 to 199 at week 20. Drop-out rates decreased from 100% in the 4th week to 35% in the 20th week, and the false positive rate was 0.07%. In 2020, Tam et al. [49] developed a system that simultaneously analyzed 356 SNPs, selected based on Asian allele frequencies, with approximately 148 markers used for CPI calculations. A notable innovation in this study was the use of Unique Molecular Identifiers during target enrichment. This molecular barcoding during sequencing helped correct PCR or sequencing errors. The authors also used a new Bayesian algorithm for the prediction of the maternal-fetal genotype and for estimating the fetal fraction for each sample. The algorithm, based on the publication of Goya et al. [65], performs iterative expectation–maximization cycles testing different fetal fractions. Only loci with a maternal-fetal genotype posterior probability ≥99.0% were used for PI calculations. Analyzing 15 maternal plasma samples (10 from the first trimester), collected between the 7th and 20th week of pregnancy, using MPS (MiniSeq^TM^, Illumina), the researchers obtained CPIs exceeding 99.9999% for all cases. Finally, in 2023, Gao et al. [59] published a new theoretical method, called Paternity Test Analysis System (PTAS) for NIPPT. This method was meant to propose a new statistical method adapted to the uncertainty of the fetal genotype. It includes four different models that allow to estimate the fetal fraction, the genotype probability, the CPI and Cumulative Probability of Exclusion (CPE), and to detect samples contamination. The method was tested on 64 early pregnancies and allowed to correctly attribute paternity in all the cases except one, for which the fetal fraction was <0.51%.

The large number of research conducted on SNPs-based method demonstrates that these markers are the most widely used in NIPPT. However, no globally accepted method has been established, and these studies reveals that there is no consensus on results interpretation.

### 3.3. Composite Multiplexes and Compound Markers

To enhance forensic genetic analyses, researchers have integrated different genetic markers within the same kit to develop composite multiplexes [52], while others have combined different genetic markers to create more efficient compound markers. Some of these approaches have shown promising results in NIPPT (Table 1).

One notable study, published by Shen et al. [52], evaluated the performance of a composite multiplex consisting of 152 markers (both SNPs and STRs) using the commercial kit ForenSeq^TM^ DNA Signature Prep (Verogen), analyzed by MPS. The study analyzed 17 plasma samples from pregnant women, collected between the 7th and the 24th week of pregnancy, with eight samples from the first trimester. The strength of this kit lies in the combination of markers that offer high discrimination power, with those targeting shorter fragments, all of which have been previously validated for forensic applications. Of the 94 SNPs tested, an average of 18 were expected to be detected in cffDNA. Results showed that, across all cases, an average of 14 paternally transmitted alleles were correctly identified, corresponding to approximately 76% of the SNPs alleles. The overall error rate (false positives) was estimated to be around 0.4%. However, for the STRs, the results were less satisfactory. For autosomal STRs, the drop-out rate reached 58%, with a drop-in rate of 16%. The analysis of Y-STRs (in two cases) and X-STRs provided insufficient information, with X-STRs exhibiting 50% false positives and a 75% stutter rate. Nonetheless, combining SNPs and autosomal STRs results significantly increased the CPIs, with 82.35% of the 17 families achieving a CPI greater than 10,000. Interestingly, this study used the R package RelMix (Available online: http://cran.r-project.org/web/packages/relMix (accessed by the authors on 14 December 2020) [66,67,68] for CPIs calculations. This software is based on a semi-continuous model and allows to infer the relationship of individuals from DNA mixtures, accounting for different parameters that could influence NIPPT results, such as drop-in, drop-out, mutations, silent alleles, and population substructure effect.

Compound markers, such as Microhaplotypes (MHs), have also shown potential for improving NIPPT. MHs are short markers (<300 bp) that combine at least two closely linked SNPs [69]. These markers retain the benefits of SNPs for cffDNA analysis while increasing their discrimination power. A power study by Wang et al. [70] demonstrated that as few as 15 MHs are required to determine prenatal paternity, with additional markers improving the accuracy. However, only a few research groups have tested the application of MHs in NIPPT. In 2020, Ou and Qu [53] reported using a 60 MHs panel, analyzed by MPS, on 15 first-trimester pregnancies. The plasma profiles were compared with the profiles of the fathers and close relatives (brothers or uncles). In all cases, the CPIs obtained with the R package RelMix, exceeded 10^12^, and the close relatives were effectively excluded from paternity. The same method was also tested on monozygotic and dizygotic twin pregnancies (*n* = 19), allowing to successfully determine paternity starting from the 10th week of gestation for all the cases [54]. Moreover, it enabled the detection of a case of pregnancy, in which two oocytes were fecundated by the sperm from two different fathers [71]. In 2022, Zhang et al. [55] developed a new panel of MHs, which was meant to be more cost-effective in comparison to MPS-based protocols. The authors used the SNaPshot technology coupled to CE, to analyze the 15 SNP-SNPs markers included in the panel. The markers set was tested on 26 plasma samples collected after the 18th week of gestation. Results showed that at least one informative allele was detected per family, with a maximum of six informative alleles per case and a detection rate of 98.7%. Despite the positive results, the authors highlighted the need to develop a larger markers system for NIPPT.

DIP-STRs [72,73] are another type of compound markers assessed for NIPPT. These markers combine two types of genetic variation: an insertion/deletion polymorphism (DIP) paired with an STR and have been initially developed to analyze mixed DNA samples with highly unbalanced two contributors ratios (up to 1:1000) [74]. Several studies have demonstrated the effectiveness of DIP-STRs in the detection of cffDNA [56,57]. The use of sequence variance in the DIP region to design allele-specific primers enables DIP-STR markers to target the minor DNA contributor in a mixture. When the paternal DIP allele transmitted to the fetus is distinct from the maternal alleles, selective amplification of the fetal DNA in the maternal plasma is possible, thereby reducing maternal DNA interference. The most recent study evaluated the performance of 47 DIP-STRs analyzed by CE, on 87 plasma samples collected throughout pregnancy, starting from the first trimester. The calculated CPI demonstrated that seven informative markers were sufficient to determine paternity. The false negative rate was approximately 6% [57]. Although DIP-STRs have already been analyzed in conjunction with other types of markers such as SNP-STRs [58] for cffDNA analysis, further investigations into these combinations would be valuable. Moreover, their analysis may be empowered by the development of large multiplexes analyzed by MPS.

## 4. Discussion and Conclusions

In this review, several genetic markers have been evaluated for their suitability in NIPPT applications, and the results reveal that an accurate protocol, particularly for the first trimester of pregnancy, has yet to be established.

While STRs are commonly used for invasive or postnatal paternity testing [75,76,77,78], they have demonstrated poor performance in NIPPT [29,30,31]. This limitation primarily stems from their relatively large size, which is not adapted to target cffDNA fragments, and from the mixed composition of maternal plasma, where cffDNA is present in low abundance. Even when mini-STRs are used to shorten the target amplicon, the predominance of maternal DNA in the samples often complicate the detection of fetal alleles [33,34]. Y-STRs, particularly Y-chromosome mini-STRs, have shown relatively good performance in plasma samples from mothers bearing male fetuses [30,37,38,39]. However, these markers cannot be used when the fetus is female. These challenges have prompted researchers to develop alternative genetic markers for improved accuracy and reliability.

The advent of MPS and its increasing use in forensic genetics have significantly advanced the development of new sets of markers. MPS has enhanced multiplexing capability and improved the sensitivity and specificity of analyses compared to CE [79,80]. This progress has been particularly beneficial for developing SNPs panels. Targeting shorter regions and being better adapted for cffDNA fragment analysis, SNPs have proven to be a valid alternative to STRs [43,44,45,47,48,49,51,59]. However, because SNPs exhibit binary polymorphism, a larger number must be analyzed to achieve the same level of discrimination power as STRs markers [61]. This implies a higher sequencing depth and, consequently, an increased cost of the analysis. Furthermore, in many cases, despite analyzing a large number of markers, only a few informative SNPs contribute significantly to the CPIs calculations, raising concerns about the cost-effectiveness of the approach. From an analytical standpoint, MPS analysis of SNPs often results in a low signal-to-noise ratio, which complicates the interpretation of results and may lead to an increased risk of false negative rates [43,45,48], especially in early stages of pregnancy where the fraction of cffDNA is lower. Additionally, some studies use markers that could be associated with genetic disorders, while others employ SNPs panels specifically developed for medical disease identification, and do not meet the established guidelines for DNA analysis in forensic contexts. Depending on the national regulations, their forensic application may rise ethical and legal concerns. Finally, although many studies on NIPPT using SNP markers have shown promising results, most of them used in-house developed panels and have been primarily validated in East Asian populations [43,44,45,46,49]. To facilitate broader application, a standardized SNPs panel validated across populations is necessary.

To address some of the limitations of SNPs, and notably their low discriminatory power, combining multiple SNPs into MHs has enhanced their variability reducing the number of markers needed for NIPPT analysis [53]. Despite the improvement, MHs analysis did not solve the problem of maternal DNA interference.

Other compound markers, such as DIP-STRs, have shown interesting cffDNA target amplification capabilities, effectively reducing the issue associated with analyzing maternal plasma DNA mixtures [56,57,81]. Similarly, new associations of markers allowing for allele-specific amplification, such as DIP-Indels [81] or DIP-SNPs markers [82], have a valuable potential. By enabling targeted amplification without generating stutter, they could be a valid alternative for NIPPT, though their performance has yet to be fully evaluated. The application of composites multiplexes revealed that simultaneous analysis of different markers types can yield better results, and lead to higher CPIs values, as compared to using one single type of marker, thereby enhancing overall performance [52].

This review also highlights the lack of standardization in methods and algorithms developed for allele calling and CPI calculations in NIPPT (Table 1). According to PTC and ISFG guidelines on best practices for reporting paternity test results, ISO17025 accredited labs should evaluate the results using the likelihood ratio (LR) approach, known as the Paternity Index (PI) [83,84]. While most of the studies have adopted these guidelines, efforts are still needed to address the particularities associated with prenatal paternity testing, including the mixed nature of plasma samples, the specificities related to each type of markers, and the technology used for their analysis. In this regard, some studies report the application of the R package RelMix [36,52,53,71], which uses a semi continuous model for CPI calculation considering some of these particularities.

Finally, better reporting methods performance, false positives and false negatives rates, is essential for accurately assessing the limitations of existing protocols and facilitate the comparisons between studies.

## 5. Futures Perspectives

Prospects include leveraging MPS analysis in the development of new multiplex assays incorporating multiple types of markers better suited for cffDNA analysis. This will allow to generate more extensive genetic data for noninvasive prenatal paternity analysis while minimizing sample consumption. Moreover, allele-specific amplification using compound markers within a single multiplex assay should be further explored. Due to their capacity in reducing maternal DNA interference, their application to NIPPT could solve several analytical challenges.

Standardization in data analysis and allele calling pipelines is needed and should consider the particularities related to the samples, the marker assay, and technology used. Moreover, the broader application of UMIs could improve the identification of amplification artifacts and facilitate the interpretation of sequencing errors, enhancing sensitivity even at low sequencing depths.

Futures directions should also include the establishment of guidelines for results interpretation in NIPPT, and the development of continuous models to support the interpretation of DNA mixtures encountered in these cases.
